# Notes and News

**Published:** 1889-08-17

**Authors:** 


### Notes and News.

COLLECTED AND COMMUNICATED.

Surely it is very childish to quarrel over the names of wards. A ward however named would do equally good work. Yet, at Hartlepool, the governors spent a whole meeting in wrangling on this subject, till someone was driven to propose numbers instead of names for the wards.

Once more we learn, and this time from the Tonga Islands, that our civilisation is a failure. The British Vice-Consul at Nuknalofa, in his last report refers to the population, and observes that although no census has been taken, it is believed that, like other South Sea aboriginal races when brought into contact with civilisation, with settled forms of government, and missionary effort, the Tongans are decreasing. A severe epidemic of a bronchial character carried off a large number in the spring of last year. They are very subject to lung diseases, and a great number die of consumption. In fact, they are fading away before the tender mercies of the white man.

The Home Secretary, upon whom a deputation of the Church of England Burial Reform Society waited, asking for an enquiry by Royal Commission into the condition of cemeteries, has, at the instance of Mr. Byron Reed, M.P., on behalf of the association, issued returns respecting the London cemeteries, by which it appears that the number of bodies at present interred in twenty-one metropolitan cemeteries is upwards of 1,276,000, and the total area covered by the cemeteries is 558 acres. In one cemetery, that of the Tower Hamlets in the East-end of London, within an area of thirty-three acres almost a quarter of a million are buried. It would be difficult to afford a better justification of the action of the association in giving the public health aspect of its work a prominent place in its programme. The movement certainly deserves the support of all social reformers, both on sanitary and on moral grounds. To educate popular opinion, however, on matters involving deeply rooted sentiment, is always a slow process. Cremation appears to be the logical future of burial reform.

The working men governors of Newcastle Infirmary seem pleased with their pet institution, which they support so heartily. The following entries have lately been made in the governors' book. On July 16, the following entry, signed by George Bell and John Capstaff: "We have this day visited the various wards, and find everything satisfactory." On July 20, the following was written by R. Clegg:- "I have been around, and am well pleased with everything, and am well pleased with the way all is arranged." On July 27, Robert Clark wrote: "I have to-day been all round the institution, and find everything to my entire satisfaction." On July 28, Robert Clark again wrote: "I have to-day again been round the institution, and find everything in good order."

Unless you are a doctor don't go to Calcutta. In the latest health report from that city Dr. O'Brien tells a strange story of 60 waggon-loads of foul refuse daily scattered over the marshes, just without the city. Dr. O'Brien says the marshes smell; or rather, being a Celt, he says "The air for miles round is poisoned with the deadly effluvia emanating from this noisome spot." If these salt-water marshes continue to be polluted in this manner it needs no prophetic spirit to predict an outbreak of plague in the city. The suburban statistics show that this system has increased the mortality in the suburbs some 20 per thousand, and the evil is increasing every day. It is intolerable that a system so dangerous to the sanitary condition of a large and wealthy city like Calcutta should be permitted to continue.

Mr. William M'Ewen writes to the *Glasgow Herald* on hospital abuses. In answer to the question, "What are we to do with our new medicos?" Mr. M'Ewen, who has had a large experience at the Royal Infirmary, says: "What are they qualified to do? having got their degree, supposed to be based on clinical instruction. The most of them have not up to that date been brought into personal contact with the treatment of disease. Few of the licentiates ever see a case of fever or measles till they have entered on their medical practice, and Dr. Allan, the accomplished physician at Belvidere Hospital, told me that, in 1885, when he established a class for clinical instruction on fever, only three out of the many students of the university attended. Last year I understand he had rather a better class, but still not one in ten looked near the hospital. If the present professors were as intent on qualifying their students as in pocketing their guineas, which, however, they have sometimes to refund, we would not have this question to answer now."

Messrs. Tinbell Brothers, of Newark, sent a thousand gallons of disinfectant to Johnstown, and in the letter from the Board of Health acknowledging their generosity, the following paragraph occurs: "As an expression of sympathy from brethren in a distant land its value is greatly enhanced. It has arrived at a fortunate time. After a month's incessant labour, often by night as well as by day, resting only on the Lord's Day, we have but just succeeded in clearing the streets of that portion of the town which was not swept away, of the tangled mass of debris, consisting of houses, passenger coaches, freight cars, locomotives, furniture, trees, bridges, corpses, and carcases, which encumbered them up to the very roofs of the houses, and once more converting them into thoroughfares. We shall be able therefore to use a street sprinkler to great advantage, and your disinfectant will come into play very acceptably in that way. Fortunately, we have been able up to the present time to maintain a very fair condition of public health, but the fervid heats are yet to come, and it will require unremitting care to prevent some serious outbreak of disease."

The following vacancies are announced this week, the amount of the salary in each case being given after the name of the institution :

**Resident Medical Officers.**

Cork Dispensary Committee. Dispenser.—£100.
Salisbury Infirmary. House Surgeon.—£100, board, &c.
Swansea Hospital. Medical Officer.—£100, board, &c.
Fulham Cancer Hospital. Two House Surgeons.—£50, board, &c.
Glasgow Victoria Infirmary. Superintendent and Medical Officer.—£125, board, &c.

**Non-Resident Medical Officers.**

Kendal Rural District. Medical Officer of Health.—£375.
Chelmsford Rural District. Medical Officer of Health.—£600.
West End Hospital, Welbeck-street. Dispenser.—£100.

Quite a convivial party met at the Convalescent Home, Matlock Bank, to celebrate the first annual meeting ; and Miss Peet, the matron, presided behind a tea-tray. The home was only opened in June, so there was not much business to transact. The treasurer announced a balance in hand, and all the former officers were re-elected.

The half-yearly meeting of the Governors of the Essex and Colchester Hospital was held in the board-room on Thursday, July 25, when Mr. Horace G. E. Green presided. The Chairman read the statement of accounts for the half-year ended June 30, which showed the receipts to be £231 11s. 10d. less than the actual expenditure. Nearly 1,000 circular letters had been sent out soliciting subscriptions and donations from persons in the neighbourhood, but they had only met with a slight response.

In reply to a question asked by Mr. Cremer in the House of Commons last week, Mr. J. W. Lowther said : "The Charity Commissioners are informed by the chapter clerk of St. Katherine's Hospital that the vacancy caused by the decease of the late master has been filled up. The duties attaching to the office are those prescribed in the rules of the hospital, which were laid upon the table of the House in the month of June last. The salary and emoluments of the new master are the same as those of his predecessor. As the hon. member was informed on the last occasion when the matter was discussed in the House, the Charity Commissioners have no power to take any steps to reform the management of the charity except upon an application from the majority of the governing body, and such application has not yet been received. The amount of salary and emoluments appears in the rules."

At the Quarterly Court of Governors of the Hospital for Consumption, Brompton, the report of the Committee of Management, read by the secretary (Mr. Dobbin), stated that since the last court the benefits of the hospital had been largely appreciated, and that in addition to the 321 beds occupied in the two buildings 120 male and female patients had been sent to seaside homes (chiefly Sandgate and Bournemouth) and maintained there at the expense of the hospital. An interesting incident had been the visit of the Princess Christian on the 15th ult., when Her Royal Highness took part in a concert to the inmates, this being the fourth occasion on which the Princess had thus gratified the patients and shown her interest in the hospital entertainments. Only one legacy had been announced—viz., that of Miss H. Stone, of £15. The committee find it needful to remind the public of the constant necessity for their liberal support, in order to efficiently maintain this useful but unendowed charity. The number of patients admitted since May 30 was 242 ; discharged, many greatly benefited, 280; died 49 ; new out-patient cases 2,301. The report was unanimously adopted, and the proceedings closed with a cordial vote of thanks to the chairman, Mr. Beckwith.

We wonder what the house physicians at the Middlesex think of Grant Allan's "The Devil's Die"? This is a nove in which one of their number is represented as committing two diabolical murders—one by the means of injecting cholera germs instead of morphia into his wife's arm. Grant Allan deals well with scientific subjects, still his knowledge of the working of hospital details is decidedly defective.

The report of the Hereford Dispensary states that the work carried on by this institution during the past year has been very satisfactory. A large number of patients have been relieved, and the income of the year has more than met the expenditure. The number of patients who brought letters of recommendation from subscribers this year again shows an increase ; 3,919 patients were relieved on application as compared with 3,547 last year. The receipts of the year from all sources have amounted to £182 2s. 7d. This includes a legacy of £30 received from the executors of the will of the late Mr. James Corbett. The total expenditure amounts to £127 6s. 2d., which, after deducting the above-mentioned legacy of £30, which has been carried to the investment account, leaves a balance of £24 16s. 5d. in the hands of the treasurer.

West Bromwich District Hospital meeting has been held : the number of in-patients admitted during the year was 556, making, with 40 remaining from last year, a total of 595, as against 494 in the previous year, an increase of 102, whilst 5,617 out-patients, of whom 2,241 were cases of accident or urgency, had been treated, compared with 4,989, of whom 1,753 were accident or urgency cases, during the previous year, this being an increase of 628. The number of out-patients treated duiing the year had been 5,617, and assuming that the out-patients cost 2s. 6d. per head, this would make a total on the cases treated £702 2s. 6d. Taking the cost of the in-patients at £2 18s. 9½d. each, a total was reached in that department of £1,751 15s. 7d. The total expenditure was £2,853 18s. 1d. The committee strongly recommend that a separate eye institution should be established, believing it would confer a great boon upon the inhabitants of West Bromwich and the district. To erect a suitable hospital that would afford accommodation for 30 beds for in-patients, with a well-designed out-patient department and administrative offices, would require an outlay of £6,000.

The *St. James's Gazette* last Saturday had an article on Pick-me-ups, which went into unnecessary details, and was less likely to act as a warning than to point out a course which some might have remained ignorant of. Customers are supposed to be seen going into a chemist's shop in the City : a customer of decidedly nervous aspect, will be treated to a morning draught of tincture of capsicum, bromide of potassium, bromide of ammonium, and sal-volatile. "A very nice nerve-restorer and brain-tonic indeed," so the chemist says; "and 'bromide' is really very harmless." With regard to the salesman's remark about the harmlessness of "bromide," it is permissible to differ very strongly from him. If we could do so, we would rather prefer to forget than to remember the fact that a man well known in London literary circles a few years ago, who was everywhere acknowledged as one of the most brilliant classics and talented journalists of the age, helped to destroy himself quite as quickly by "bromide" as ever he did by careless living. Another popular pick-me-up—liquor strychnia, tincture of orange, and disulphate of quinine. And strychnine, so you are told, must be harmless, for certain pick-me-up drinkers can take ten grains at a time. We cannot help people from taking poison in small doses save by warning them of the insiduous nature of pick-me-ups, and pointing to the many tragedies which might have been averted if evil habits had never been begun.

